# Be a burden

**DOI:** 10.1017/S1478951524001664

**Published:** 2024-11-12

**Authors:** Benjamin W. Frush

**Affiliations:** Kennedy Institute of Ethics, Georgetown University, Washington, DC, USA

One of the fundament skills emphasized during palliative care training is learning to elicit the goals and values of those we care for. What I have learned through asking this question repeatedly and in varying ways this year is that many patients struggle to articulate those values, goals, or wishes that they hold as most important. This is understandable, as it is a question most of us are not used to facing, and likely have not considered ourselves. Yet amidst this inarticulacy, I have also learned that many patients *are* able to definitively share what they would choose to avoid, providing an answer that has proved remarkably consistent across a diverse patient population: that is, to avoid becoming a “burden,” or dependent on others.

In palliative care training, we are taught that the purpose of our work is to elicit and help pursue that which is most important to our patients. Yet as I have heard this response repeatedly expressed this year, this aversion to dependence and fear of becoming a burden, I have come to question whether affirming this desire is in fact in our patients’ best interests. Indeed, what might it mean to instead explore and even challenge this fear – to reconsider what it means to be a burden?

This may seem like a strange, even objectionable message to convey. Indeed, on the face of it, the desire to be independent, self-sufficient, and productive seems laudatory, even arguably reflecting our country’s founding ethos. Like my patients who fear dependence, I also value my ability to attend to my own physical needs and to provide for myself; to a significant degree I associate this sense of agency with my identity. Yet there comes a point where the valuation of this identity becomes counterproductive, and even harmful, for both our patients and ourselves.

One of the most important benefits of the disability rights movement in our country has been its ability to identify and challenge the extent to which we in American society assume a certain anthropology, or view of what it means to be human, that is both false and destructive. Our culture increasingly views certain characteristics as normative and desired – those of independency, agency, and productivity – while implicitly devaluing those who are more overtly dependent (Snead 2020).

The damaging effects of such an anthropology are not confined to the disability community. This privileged understanding of what it means to be human has permeated society at large, including the patients we serve, and can lead to unrealistic expectations of our experiences in health, and especially in sickness. At its core, this vision of human flourishing neglects the significant extent to which all of us are constitutively dependent upon others, from cradle to grave. This dependence may prove easier to shield in moments of relative youth, health, and prosperity, but will overtly manifest for all of us at some point.

The moral philosopher Alasdair MacIntyre echoes this point in his book *Dependent Rational Animals*, arguing that to fail to recognize the communities upon which depend – and even “burden” at different points – is to fundamentally misunderstand our human condition. As I see in the patients I often care for, this reality manifests especially starkly as we age and move forward to varying degrees of senescence, or fall victim to illness. It is for this reason that MacIntyre writes, “It is important to remember that there is a scale of disability on which we all find ourselves,” (MacIntyre 1999).

It is important to note the medical system’s complicity in this view of human identity and worth that we often reinforce in our interactions with patients. To lionize independence and self-sufficiency as definitive of what it means to be human has led us to pathologize aging, a constitutive (not pathological) human experience. This is evident from the fact that we can deem “aging” or “old age” a billable problem to physicians’ explicit association of “successful aging” with maintaining independence in all facets of life, implying a level of agency we simply do not possess as we grow older (Moyse 2022). This tendency, to the degree that it associates maintenance of independence with health, also runs the risk of denigrating the basic (and critical) human response to rely upon others in times of sickness – times when it should be viewed as reasonable to become a “burden.”

It is for this reason that we should reexamine the stigma associated with terms like “dependence” and “burden,” as these states (even if tacit in times of youth and good health) reflect a reality about our human condition rather than an exception. As we fall ill or age, it is a norm, not a failure, that we would draw upon the communal networks we have spent our lives cultivating. MacIntyre would go so far as to say that this represents a healthy and virtuous response; that we as individuals and as a society must work to develop what he terms “virtues of acknowledged dependence” – such virtues which include just generosity, hospitality, and “misericordia” – the ability to take on the suffering of another as one’s own – which manifest most clearly in seasons when our vulnerability is accentuated (MacIntyre 1999).

Therefore as clinicians, to the degree that we are able, we ought to respectfully challenge patients’ fears of becoming progressively dependent, or even a burden upon others, as they age or progress in their illness. At the very least, we ought to assuage the undertones of guilt that often accompany these statements, and remind them that the prospect of dependency they face is one that we all will encounter. We ought to explore the networks of community that patients can draw on and remind them that, as the theologian Dietrich Bonhoeffer once wrote, “only as a burden is the other really a brother or sister and not just an object to be controlled,” such tendency toward objectification and control we too often fall prey to as clinicians when interfacing with sick or aging patients (Bonhoeffer 1996).

None of this is to suggest that we minimize, dismiss, or patronize patients’ fears of what it means to lose certain faculties in the course of their aging or illness. Moreover, amidst our cultural epidemic of loneliness, it is important we not resort to glib truisms, particularly when patients do not have robust communities to fall back upon. These conversations must take into account the frank difficulties associated with the loss of agency patients once experienced, which can be an occasion for mourning – the “misericordia” MacIntyre mentions as an essential virtue of acknowledged dependence.

Yet such mourning ought also make space for the opportunity present in such situations for our patients to flourish even in dire circumstance – to depend upon those connections that constitute us as communal beings, to bear on our friends, families, and broader networks who remain steadfast. For it is only in these moments of need that we recognize that sickness and senescence, far from robbing us of who we are, actually provide an occasion to display what it means to be human – to have the courage to receive.
